# Applications and interventions of polymers and nanomaterials in alveolar bone regeneration and tooth dentistry

**DOI:** 10.1039/d4ra06092j

**Published:** 2024-11-12

**Authors:** Prashish Sharma, Sushmita Saurav, Zeba Tabassum, Bhawana Sood, Anil Kumar, Tabarak Malik, Anand Mohan, Madhuri Girdhar

**Affiliations:** a School of Bioengineering and Biosciences, Lovely Professional University Phagwara 144401 Punjab India; b School of Physical and Chemical Engineering, Lovely Professional University Phagwara 144401 Punjab India; c Gene Regulation Laboratory, National Institute of Immunology New Delhi 110067 India; d Department of Biomedical Sciences, Institute of Health, Jimma University Jimma 0000 Ethiopia tabarak.malik@ju.edu.et; e Division of Research and Development, Lovely Professional University Phagwara 144401 Punjab India madhurigirdhar007@gmail.com

## Abstract

Inflammatory diseases exert a significant influence on the periodontium, serving as a primary contributor to the development of periodontitis. The advancement of periodontitis, characterized by manifestations, such as gingival recession, increased periodontal pocket depth and resorption across the alveolar bone, cementum and periodontal ligaments, poses a significant risk of dental detachment. Untreated or delayed treatment further worsens these deleterious outcomes. This emphasizes the critical importance of timely and effective interventions in reducing the consequences associated with periodontitis. Addressing these challenges requires to focus on the fabrication of bioactive materials, particularly scaffolds, as pivotal elements in tissue engineering processes aimed at alveolar bone regeneration. The incorporation of natural polymers, particularly their amalgamation with clays and clay minerals, such as montmorillonite and LAPONITE®, has been identified as a prospective pathway for advancing biomaterials in the realm of dentistry. This amalgamation holds significant potential for the production of biomaterials with enhanced properties, underscoring its relevance and applicability in dental research. This review paper explores the current advancements in natural polymer-based biomaterials employed in various dental applications, including oral caries, regenerative medicine and alveolar bone regeneration. The principal aim of this investigation is to briefly compile and present the existing knowledge while updating information on the utilization of natural polymers in the formulation of biomaterials. Additionally, the paper aims to elucidate their applications within contemporary research trends and developments in the field of odontology. This article extensively delves into pertinent research to assess the progress of nanotechnology in the context of tissue regeneration and the treatment of oral diseases.

## Abstract 2

Inflammatory diseases exert a significant influence on the periodontium, serving as a primary contributor to the development of periodontitis and alveolar bone loss.

## Graphical abstract

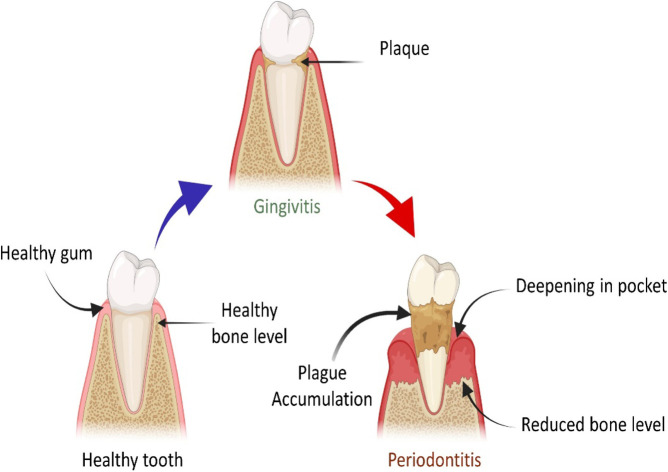

## 1 Introduction

Loss of bone leads to periodontal condition of teeth, complicates implant placement, and in severe cases, may end up in noticeable changes in face morphology. Regeneration of the tissues through the process of bone substitute implantation is a commonly employed technique to address this issue. In the United States, approximately 500 000 implanting of bone processes are carried out annually in medical and dental offices.^1^ Bone tissue's dynamic nature allows it to adapt to environmental signals through a continuous process of reabsorption and synthesis, regulated by numerous local and systemic molecules that govern bone homeostasis. The mechanical stresses can reinforce bone to maintain optimal shapes, while exposure to chronic stresses may trigger fracturing along with other damages to the bone, which may commence the process of healing.^2^ The process involves three sequential procedures: initiation of the inflammatory response, subsequent bone formation and eventual bone remodelling. A surplus of cells and molecules participate in each of these phases. The jaw bones, unlike other bones, possess unique and distinctive qualities, characterized by their uneven structure. The majority of research in this area focuses on longer bones. Hesse *et al.* reported that the tibia has a substantially higher bone mineral density than that of the jawbone, indicating that the jawbone undergoes rapid bone regeneration.^3^

Furthermore, appropriate mastication extensively promotes jawbone renewal to mitigate microdamage to the bone. In spite of that, a number of investigations have documented that distinct osteogenic abilities in mesenchymal stem cells prevail from the mandibles, in contrast to those churn out from the long bones. Preimplantation evaluation of the jawbone anatomy is also vital, and any reforms could negatively affect the tissue regeneration ability and the mandibular vascular supply. These details are crucial when considering regenerative medicine for the mandibular bone, as it has unique and distinctive characteristics compared to other similar bones. The gold standard for the self-generated grafted bone for jawbone regeneration is either non-vascularized or vascularized. The fibular free flap is very prominent, because it is thought to be the most donor bone in the human body.^4^

**Fig. 1:**
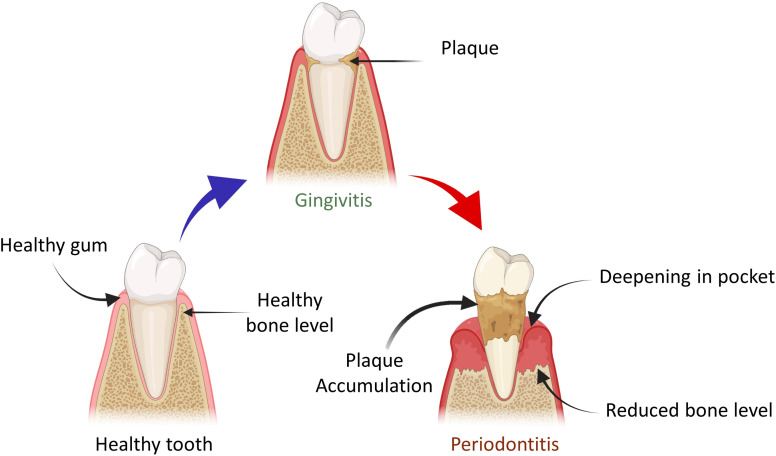
Demonstration of the transition of healthy tooth into periodontal disease and plaque accumulation.

Alveolar bone loss can be due to various causes, the most prominent pathogenic factor being periodontitis shown in Fig. 1, but it can also be caused by trauma, orthodontic therapy, tooth extraction and hormonal imbalance.^5^ Clinical observation has led to the classification of alveolar bone loss in two major types: horizontal bone loss (supra bony defect) and vertical bone loss (infrabony defect).^6^ When vertical bone losses arise in an oblique direction, an infrabony periodontal pocket is left behind. When bone losses occur horizontally, the remaining bone edges become nearly perpendicular to the tooth surface, causing a suprabony deformity.^7^ In particular, the most prevalent type of bone defect seen in clinical practices is horizontal alveolar bone loss. In the mandible, posterior and anterior sections are common locations for horizontal bone loss. The molar or premolar areas are typically where vertical bone loss arises. Furthermore, it has been revealed that there is a correlation between the range of vertical defect and the angel of horizontal defect.^8^ From a biological standpoint, periodontal regeneration includes the restoration of the damaged root surface and alveolar ridge, and they are joined by periodontal ligament (PDL). The periodontal ligament plays an imperative role in regeneration and repair processes. The integration of collagen fibres on exposed root surfaces maintains stability between the cementum and the alveolar bone.^9^ For the practitioner specializing in periodontal and maxillofacial surgery, the augmentation and reconstruction of the alveolar bone are challenging and complex areas. The primary objective for the diagnostics in this domain is to elevate the proportion of bone density in patients, who have undergone tissue loss, under certain conditions including congenital defects, aging, trauma, neoplastic pathology, periodontal disease and reconstructive surgery.^10^ These days, the most common and acceptable procedure is auto-transplantation from the intra- and extra-oral donor region of the patient. The donor site morbidity, accompanying pain, restricted supply and inappropriate blood circulation of grafted tissues are the major drawbacks of this traditional method. All these factors usually limit the use of autologous grafting.^4^ However, autologous grafting has several disadvantages such as risk of transferring infection from donar to the recipient and further rejection of the graft. Artificial alloplast is another method for bone graft composed of metals, ceramics and polymers. Some drawbacks of alloplasts include non-optimal assimilation into adjacent tissues in the damaged region and the prospect of failure owing to infection after implantation. Furthermore, their applicability is restricted to areas with elevated mechanical stress or strain.^11^ Due to the certain downsides and limitations of bone grafts in the past few decades, numerous cutting-edge methods that include tissue engineering and regenerative medicines are the emerging alternatives to the traditional procedure. The principal idea behind regenerative and tissue engineering is to use scaffolds in combination with cells, growth factors and gene delivery or use scaffolds alone to form tissue engineering constructs, which stimulate tissue repair/regeneration.^12^ The use of scaffolding materials is influential for tissue regeneration as they dispense the microenvironment that is competent for adhesion of cell, cell proliferation and cell differentiation. To mimic the extracellular matrix structure of the original tissues, the exemplary scaffolding material should have biocompatibility and biodegradability and also possess physio-chemical characteristics.^13^

This review paper highlights the recent advancements in natural polymer-based biomaterials used in various dental applications such as treating oral caries, regenerative medicine, and alveolar bone regeneration. The main objective is to summarize and update existing knowledge on the use of natural polymers in biomaterial formulations. Additionally, the paper aims to clarify their applications within current research trends and developments in odontology. It thoroughly examines relevant research to evaluate the progress of nanotechnology in tissue regeneration and the treatment of oral diseases.

## 2 Natural polymers

A myriad of natural biopolymers exist, including carbohydrate-based materials such as plant-based cellulose, bacterial cellulose, and chitosan, as well as protein-based biopolymers such as silk, albumin, keratin, and collagen.^14^ Bio-based polymers and their composites have a wide range of applications across various fields. In packaging, they provide sustainable alternatives to traditional plastics, offering biodegradability and enhanced properties. In tissue engineering and regenerative medicine, these materials are used for bone regeneration, dentistry, and drug delivery due to their biocompatibility and tailored functionality. Additionally, bio-based polymers contribute to environmental protection by aiding in pollution degradation, showcasing their versatility and potential in addressing diverse industrial and medical needs.^15–19^

Synthetic polymers have found extensive applications across diverse biomedical contexts.^20^ The mechanical attributes inherent to synthetic polymers renders them appealing for diverse biomedical applications. However, the remarkable absence of bioactive elements, specifically limited cell-anchoring sites in synthetic polymers, presents a substantial hurdle in tissue engineering. This limitation impedes the facile proliferation, differentiation and cell migration within such matrices. The integration of natural biopolymers *via* chemical modification within synthetic polymers enables their conversion into bioactive compounds, yielding biocompatible and functional materials, which mimic the native environment and enhance cell biological activity.^21^

### 2.1 Bacterial cellulose (BC)

Cellulose is a widely prevalent natural polymer derived from the cell walls of plant and synthesized by some specific strains of bacteria.^22^ Cellulose is composed of units comprising d-glucopyranose connected *via* glycosidic bonds of β-1,4 configuration. Bacterial cellulose has emerged as a compelling biomaterial in recent years, attracting attention for advanced medical applications such as scaffold and implants within the domain of tissue engineering.^23^ The molecular formula of bacterial cellulose resembles that of the plant cellulose, represented as (C_6_H_10_O_5_)_*n*_. Despite this similarity in molecular structure, a distinct divergence is observed in several physical and chemical properties. These differences manifest in areas such as purity, ultra-thin structures, as well as mechanical strength, crystallinity and a notable difference in water-retention capacity.^24^ Bacterial cellulose is synthesized *via* the metabolic activities by an obligate aerobic bacterium, primarily attributed to the *Komagataeibacter* genus. The predominant contributor is *Komagataeibacter xylinus*, previously known as *Acetobacter xylinum* and *Gluconacetobacter xylinus*.^25^ One advantageous aspect of employing bacterial cellulose as a biomaterial lies in its versatility in form and structure, allowing for the creation of diverse shapes featuring an adaptable three-dimensional (3D) network structure characterized by substantial and interconnected porosity. Bacterial cellulose also exhibits favourable biocompatibility and mechanical properties comparable to those of various native tissues, both hard and soft, making it particularly favourable choice in the field of biomaterials.^26,27^

#### 2.1.1 Synthesis, structure and properties of bacterial cellulose

Cellulose, a polymer exhibiting linear structure, is composed of anhydro-d-glucose units linked by β-1,4-glycosidic bonds. The intermolecular interactions involve numerous H-bonds formed between the hydroxyl functional groups of adjacent chains of glucose.^28^

The interaction between cellulose chains enables various polymorphic conformations such as cellulose I (characterized by a parallel chain arrangement) and cellulose II (marked by an antiparallel chain orientation), with the latter being thermodynamically more stable.^29^ The characteristic of insolubility transforms bacterial cellulose into a biostable and non-resorbable material. This characteristic makes BC particularly advantageous in dental and oral applications. Examples of such applications include guided regeneration and dental pulp regeneration in periodontal treatments.

Modification in microarchitecture is a crucial consideration in bacterial cellulose production, influenced by the conditions of culture medium. Specifically, the introduction of carboxymethylcellulose has been observed to elevate the viscosity of the culture medium, subsequently impacting the characteristic of bacterial cellulose materials. This alteration is manifested through a reduction in fibre diameters and increased pore sizes. Such variations in microarchitecture underscore the significance of optimizing culture medium conditions to attain desirable features in bacterial cellulose production.^30^ An additional illustration of scaffold modification is the particle leaching method, which can be employed to adjust the permeability of bacterial cellulose biomaterials. Certain researchers have successfully engineered scaffolds with precise nanoscale porosity by introducing particles composed of paraffin-based wax and starch of varying dimensions into a bacterium cultivation environment. Further investigations have assessed different significant factors and growth conditions that influence BC production, which, in turn, determine its unique characteristics and properties. The versatility of bacterial cellulose for its high production capacity provides the ability to attain diverse shapes and controlled microstructures such as fibre diameters and matrix porosity. These remarkable facilities for its deliberate design of novel biomaterials enrich with customized properties, presenting considerable promise for use in various oral and dental contexts.^29^ Bacterial cellulose, characterized by its metabolic inertness and exceptional purity at the nano-fibril level, serves as a biomaterial with notable applications in oral and dental tissue engineering. When primarily used for periodontal regeneration, dental pulp regeneration and as a dressing for surgical wounds in the oral mucosa, bacterial cellulose demonstrates its efficacy. Additionally, it is applied in dental root canal therapy, demonstrating its potency in the comprehensive elimination of residues and subsequent drying of the canal.^31^

#### 2.1.2 Applications of bacterial cellulose in odontology

**Fig. 2:**
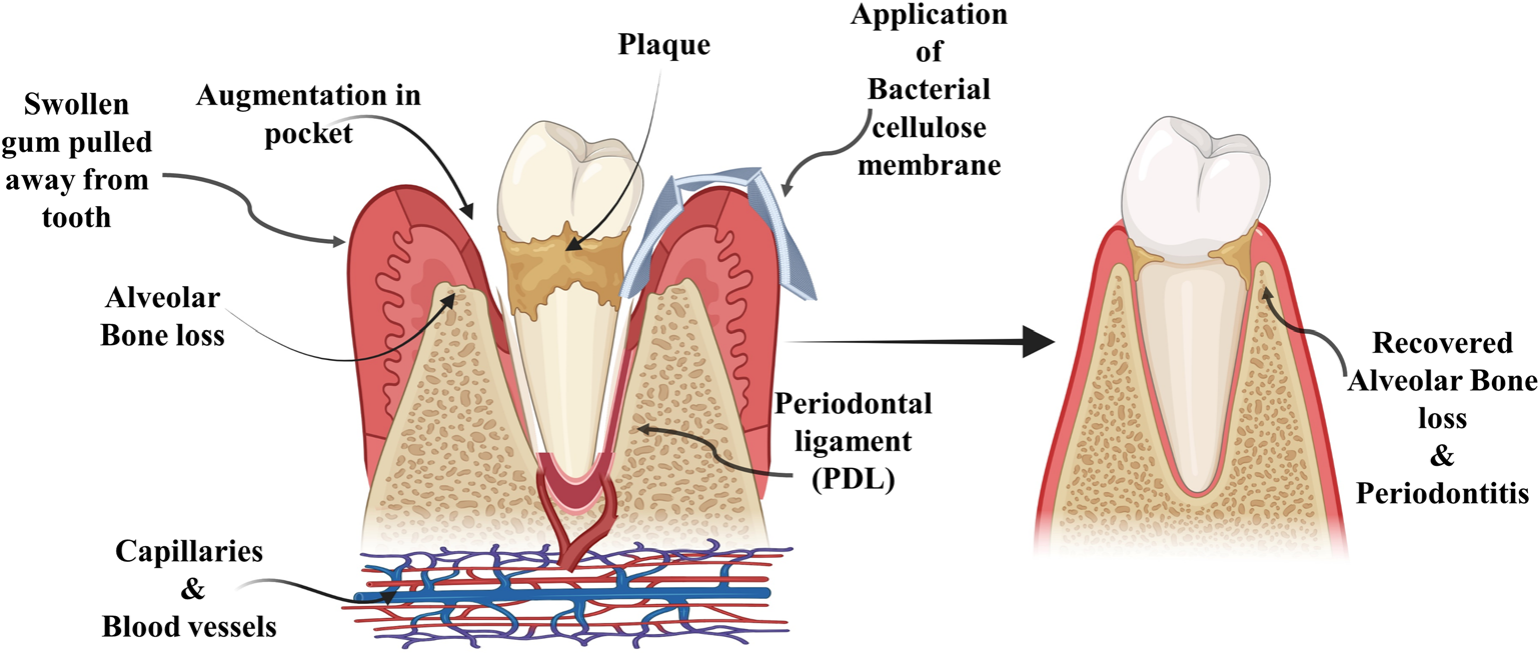
Bacterial cellulose membrane in periodontal disease and alveolar bone loss.

In the realm of dentistry, materials based on bacterial cellulose (BC) remain relatively underexplored, despite their considerable potential in tissue engineering and biomedicine. Limited studies have been conducted to illustrate the application of BC in oral and dental applications, as shown in Fig. 2. Among the initial applications in dentistry, the focus has been on the guided tissue regeneration (GTR) methodology for the treatment of periodontal diseases. The fundamental principle of GTR involve the strategically placed physical barriers at designated locations to impede the downward movement towards the apex of gingival and connective tissues of epithelial cells. This, in turn, creates a restricted region conducive to the migration of periodontal ligament (PDL) cells and mesenchymal cells for the promotion of periodontal regeneration over exposed root surface.^32^

An investigation conducted by An and colleagues involved the fabrication of bacterial cellulose membranes (BCMs) for the purpose of evaluating guided bone regeneration (GBR) in rats. Electron beam-irradiated bacterial cellulose membranes underwent comprehensive characterization through techniques such as Scanning Electron Microscopy (SEM), wet tensile strength measurement, Thermogravimetric Analysis (TGA) and Attenuated Total Reflectance-Fourier Transform Infrared (ATR-FTIR) Spectroscopy. Additionally, *in vitro* analysis was performed to ascertain cytocompatibility. This collective finding from mechanical, chemical and biological assessments revealed effective interactions between EI-BCMs and cells, ultimately fostering bone regeneration.^33^

Conversely, addressing surgical wounds within the oral mucosa represents a significant concern in dentistry. Minor defects of limited extent typically undergo healing themselves without much trouble. However, in cases of moderate defects, split or full-thickness grafts may be employed. Notably, for defects encompassing a substantial portion of the buccal mucosa, a secondary surgical intervention becomes imperative.^32^

Dental implant procedures have become common place in clinical practice and it is not unusual for dentists to encounter challenges related to insufficient bone height in the upper jaw region.^34^ Koike and group conducted experiments involving frontal bone defects in a cohort of 12 rabbits, categorized into four groups: BC (Solely subjected to BC grafting), BMP-2 (exposed exclusively to bone morphogenetic protein-2 solution) and bacterial cellulose + bone morphogenetic protein-2 (bacterial cellulose augmented with bone morphogenetic protein-2 graft). The findings revealed that BC effectively retained the graft space, facilitating the controlled release of BMP-2 into the targeted region. This underscores the potential of bacterial cellulose + bone morphogenetic protein-2 as a viable choice for enhancing the structure of bones and facilitating the insertion of dental implants.^35^

Voicu and team have recently developed a noble series of nanocomposites using bio-cellulose membranes and mineral binding powders, and demonstrated that these nanocomposites pose significant potential in the field of endodontics. The nanocomposites were prepared by mixing silicate cement through the sol–gel technique, wherein a BC polygranular powder, obtained through hydrothermal treatment and subsequently crushed into micrometric particles, was incorporated. The resulting nanocomposite underwent comprehensive analysis through X-ray diffraction, scanning electron microscopy, thermal assessments and various mechanical assessments. By the use of bacterial cellulose it accelerate the process which can lead to shorter visits for treatment and reducing the overall time needed for care. *In vitro* assessments were conducted, revealing that all materials demonstrated non-cytotoxic effects, fostering cell adhesion and proliferation. Additionally, these materials exhibited a significant mineralization process under a simulated environment. The researchers concluded that the suggested composite holds substantial capacity for a magnitude of application in endodontic therapy, particularly in the realms of facilitating dentin remineralization, addressing root perforation and root canal filling.^36^ Concerning reinforcement properties, BC demonstrated favourable outcomes by enhancing mechanical attributes when amalgamated with chitin fibres for applications in surgical sutures. This establishes BC as a compelling contender for the advancement of bacterial cellulose-based medical sutures in dentistry.^37^

Coelho and colleagues innovatively synthesized a biomaterial comprising a bacterial cellulose membrane combined with hydroxyapatite in conjunction with an antibody directed against bone morphogenetic protein “(anti-BMP-2)” “(BC-HA-anti-BMP-2)”. The confirmation of BC and hydroxyapatite presence was achieved by FTIR spectroscopy and SEM-EDS. The biomaterial demonstrated an upregulation in the gene expression associated with bone repair. Furthermore, it exhibited genotoxic, non-cytotoxic and mutagenic characteristics evaluated with MC3T3-E1 cells.^38^ Bacterial cellulose stands out as a flexible, mucoadhesive and soft material, rendering it highly suitable for various dental applications. Its versatility allows for utilization as a wound dressing on the palate for mucosal graft, a covering for extraction sockets^39^ and a temporary implant in dental extraction sites or a membrane for guided tissue regeneration.^40^

### 2.2 Chitosan

Chitosan is one of the most propitious biomaterials used in dentistry. The derivatives of chitosan have outstanding biodegradability, antimicrobial activity, and reactivity of deacetylated groups.^41,42^ They have the ability to form sponge, gel and films. High bioactivity of the chitosan is the most dominant quality that make it worthy for the preparation of biomaterials for dentistry.^43^ Chitosan is a cationic linear chain polysaccharide biopolymer. It is the deacetylated form of chitin, which is a natural polysaccharide obtained from the cell walls of fungi such as *Mucor* and *Aspergillus*, and the exoskeleton of crustaceans such as prawns, insects, and shrimps.^44^ The extent of deacetylation indicates the proportion of free NH_2_ groups in chitosan. The average quantities of 2-acetamide-2-dexoy-d-glucopyranose and 2-amino-2-deoxy-d-glucopyraose units were determined by the average acetylation degree of chitosan. In addition to having significant impacts on the chemical and biological properties, the relative percentage of these units affects chitosan's solubility.^43^

#### 2.2.1 Structure and properties of chitosan

**Fig. 3:**
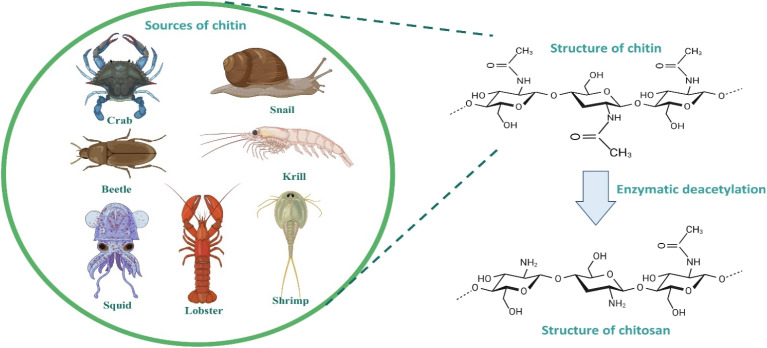
Sources and structure of chitin and chitosan.

Chitin and chitosan possess unique physical, chemical, and biological characteristics, making them highly valuable for applications in different fields. They are the second most prevalent type of polysaccharides on the Earth. Chitosan is produced through the deacetylation of chitin, resulting in a structure where *N*-acetyl-d-glucosamine and d-glucosamine are linearly polymerized.^45^ Morphologically, chitosan exists in numerous forms including crystalline, semicrystalline, and unstructured. Depending on the source of chitin, the molecular weight of chitosan ranges from 300 to 1000 kDa. The deacetylation of chitosan from chitin and its different sources is depicted in Fig. 3. The molecular formula is represented as (C_6_H_11_NO_4_)_*n*._^46^ The deacetylation process of α-chitin involves the removal of acetyl groups, leading to the generation of amino groups and ultimately yielding chitosan. For every monomer, it has two hydroxyl groups and one primary amine. The amine group on the deacetylated unit and the repetitive primary and secondary hydroxyl groups give chitosan its chemical activity. A free amino group is present in d-glucosamine and it absorbs a positive charge. These characteristics include solubility and antibacterial activity.^47^

In the solid state, chitosan manifests as a semicrystalline polymer, exhibiting a white or faintly yellow appearance. Within this state, the chitosan molecule organizes into crystallites of significant order, characterized by the presence of two predominant crystalline polymorphs. The inherent crystallinity of chitosan renders it amenable to interactions with various reagents.^48^ Notably, chitosan exhibits non-thermoplastic behaviour, undergoing degradation prior to reaching a melting point. It is insoluble in water and most organic solvents, yet demonstrates solubility in aqueous acid solutions, including formic acid, lactic acid, citric acid and acetic acid, as well as in solvents such as dimethyl sulfoxide and *p*-toluene sulphonic acid. The complexity of chitosan mandates the development of a precise method for evaluating its physiochemical properties.

The characteristics of chitosan are significantly impacted by both its molecular weight and the degree of deacetylation. Notably, chitosan with an elevated molecular weight demonstrates superior elongation and stability to chitosan with lower molecular weights. Moreover, membrane crystallinity and molecular interactions are intensified in high-molecular-weight chitosan as opposed to low-molecular-weight chitosan. Chitosan is regarded as a weak base since it dissolves in aqueous solutions. This characteristic arises from the protonation of the amino group distributed within the chitosan molecule, resulting in substantial charge density, equivalent to one cationic charge per glucosamine unit. Dilute inorganic acids, with the exception of sulfuric acid, serve as appropriate solvents of chitosan, while a nonpolar organic solvent is ineffective in dissolving it. Prior to reaching the glass transition phase, the thermal degradation of chitosan occurs.^49^

#### 2.2.2 Application of chitosan in dental implants

The significance of coating and surface treatments in the context of dental implants is paramount for facilitating the osseointegration process. Chitosan, recognized for its remarkable metal-binding capability, presents a promising way to enhance the durability and mechanical strength of titanium implants. However, the solubility of chitosan in aqueous solutions depends upon the pH environment. At neutral pH, a notable portion of chitosan molecules undergoes deprotonation, leading to the formation of solid particles from the solutions. The adhesion strength of chitosan coatings on titanium surfaces expresses reservations about their appropriateness for clinical applications. The compressive examination of chitosan as a titanium coating has been a subject of thorough investigation by various researchers. However, a limited number of *in vivo* studies have been disseminated in the literature.^50^ The clinical efficacy of dental implants hinges on the extent of osseointegration between the implant materials and the alveolar bone. When the coating of chitosan is applied to the interface between the bone and the implant surface, it causes several changes in the mechanical, biological and surface properties of the implant.^51^

#### 2.2.3 Applications of chitosan in periodontal regeneration and oral drug delivery

As a chronic inflammatory condition, periodontitis is triggered by infections caused by bacteria, impacting the structures that support teeth and resulting in substantial deterioration of the periodontium, encompassing the alveolar bone, cement, gingiva and periodontal ligaments. This pathological condition is prevalent worldwide, affecting more than half of the population in the United States and standing as a primary etiological factor contributing to the loss of tooth. In certain instances of increased severity, the emergence of infrabony defects is distinguished by the formation of periodontal pockets. Addressing these lesions poses a great challenge for clinicians in this field.^52^ Undoubtedly, the primary objective of periodontal intervention is to mitigate inflammation and manage infection through a combination of chemical modalities (such as antiseptics and antibiotics) and mechanical treatments (including root planning and scaling). However, this therapeutic approach is predominantly aligned with wound repair, marked by the development of an extended junctional epithelium. In cases where there is significant severity, especially when the pocket depth exceeds 5 mm, depending solely on non-surgical treatments might not be enough, emphasizing the necessity for additional therapeutic approaches.^53^ Over the past decade, considerable attention has been paid to the development of chitosan-based delivery systems.^54^ Various devices incorporating chitosan have been formulated and assessed within distinct contexts, encompassing gels, fibres, micro/nanoparticles and membranes.^55^ Certainly, empirical evidence has demonstrated that chitosan-based gels, specifically within the concentration range of 1–4%, exhibit favourable viscosity suitable for injecting into periodontal pockets. Significantly, these gels serve as dependable carriers for the targeted release of active pharmaceutical agents at the site of disease. While achieving a sustained release that is considered to be ideal, it is crucial to recognize that the release kinetics is a parameter linked to the amount of chitosan present.^56^ Pharmaceutical agents, including statins,^55^ doxycycline,^57^ and alternative antibiotics/antiseptics such as tetracyclines have been integrated in such devices.^58^

Chang and co-workers conducted an *in vivo* assessment using an experimental murine model of periodontitis to examine the application on hydrogels made up of chitosan incorporating naringin, which showed anti-inflammatory properties and its compound originated from nature. This investigation elucidated the prominent characteristics of the chitosan hydrogel, revealing that its administration at the site of the lesion resulted in the acute phase of inflammation by the release of active compounds. This release mechanism induced the effect of anti-inflammation amid the periodontal tissues.^59^ The observed outcomes could be rationalized through the controlled administration of the active agent at the lesion site and the three-dimensional attributes inherent to the chitosan scaffold. In particular, this study also illustrated that chitosan has the capability to enhance the antibacterial efficacy of chlorhexidine.^60^

#### 2.2.4 Application of chitosan in dental pulp regeneration

Scaffolds constitute a pivotal component in endodontic treatment and play a vital role in the delivery of active molecules and its transportation of proficient cells within the endodontic compartment. Recently, scaffolds based on chitosan have been engineered for these specific purposes.^61^ These scaffolds prove advantageous not only for dentin formation but also for pulp regeneration ability, and induced mineralization. This scaffold composed of chitosan incorporating β-tricalcium phosphate (β-TCP) has exhibited a significant upregulation of mineralization markers, alkaline phosphatase and osteopontin, fostering the formation of dentin by human periodontal ligament cells.^62^ Moreover, recent findings underscore that a scaffold composed of chitosan stimulates the proliferation of cells, migration of mesenchymal stem cells and odontoblastic differentiation of dental pulp stem cells, both *in vivo* and *in vitro*.^63^ The antimicrobial properties of chitosan have been applied in various fields, resulting in significant findings.^64^ The endodontic space, located at the centre of tooth, comprises the vital pulp, which is essential for maintaining tooth vitality. Including the soft connective tissue, this pulp also contains vascularization, dental stem cells, innervation and collagen fibre, all enveloped by dentin. This dentin–pulp complexes, characterized by their high reactivity, tend to exhibit a challenging and prolonged inflammatory response following substantial aggression, and this inflammatory reaction often proves difficult to alleviate, culminating in pulp necrosis.^61^ Traditional endodontic procedures are extensive, involving the removal of all tissues, irrespective of their regenerative potential. In recent times, regenerative approaches have emerged, targeting the restoration of vascularization dentin, radicular structure, innervation and pulp connective tissues. Scaffolds play a central role in endodontic treatment by facilitating the delivery of transporting competent cells and active molecules across the endodontic compartments. Scaffolds possess suitable consistency to enable their injecting throughout the root canal system and their porosity is of paramount importance for effective cell colonization.^65^

### 2.3 Polyhydroxybutyrate (PHB)

Microorganisms have the capacity to serve as production hubs for transforming a range of nitrogen and carbon sources into a diverse array of extracellular and intracellular biopolymers. This includes polyhydroxyalkonates (PHAs) and exopolysaccharides (EPS), and these conversions occur under distinct stress conditions.^66^ Polyhydroxyalkonates (PHAs) function as internal repositories for carbon and energy storage within microorganisms.^67^ PHB is a specific type of PHA with distinct properties. Recent reports have identified a diverse range of microorganisms capable of producing polyhydroxyalkonates (PHB), including but not limited to *Pseudomonas resinovorans*, *Halomonas cerina*, *Bacillus mycoides* and others.^68^ Galactose, present in the biomass of marine algae, was investigated by Jung *et al.* conducting screenings on 16 distinct strains of *Halomonas*. Their findings highlighted a notable production of polyhydroxybutyrate (PHB), *Halomonas cerina* reaching a concentration of 5.2 g L^−1^ using *Eucheuma spinosum* hydrolyzate as a source of carbon. Notably, *Halomonas cerina* exhibited resilience in a high-saline environments and demonstrated the ability to accumulate PHA under non-sterile conditions up to 72.41% w/w.^69^ In a separate investigation, scientists employed a cardboard hydrolysate as a source of carbon for *Bacillus mycoides*, noting a substantial accumulation of polyhydroxyalkonates (PHAs) amounting to 56% w/w.^70^

Bacteria with the capability to produce natural poly(3-hydroxyalkonates) (nPHAs) store this polymer as distinct inclusions, commonly referred to as granules, within the cytoplasm. These granules typically exhibit diameters ranging from 100 to 800 nm. Particularly, symbiotic and pathogenic bacteria associated with human environments, such as *Clostridium*, *Bacillus*, *Vibrio*, *Pseudomonas*, *Acinetobacter*, *Fusobacterium*, *Streptomyces*, *Rickettsia*, *Agrobacterium*, *Ralstonia*, *Burkholderia*, *Legionella*, *Mycobacterium*, *Sphingomonas*, *Neisseria* and *Bordetella* possess the capacity to synthesize Polyhydroxybutyrate (PHB). Additionally, these bacteria either produce the enzymes responsible for PHB biosynthesis or harbour the corresponding genes, primarily those encoding PHA polymerase. Certain bacteria, for instance, *Pseudomonas* species, demonstrate the ability to synthesize both PHB and its various copolymers.^71^

#### 2.3.1 Structure and properties of polyhydroxy butyrate

**Fig. 4:**
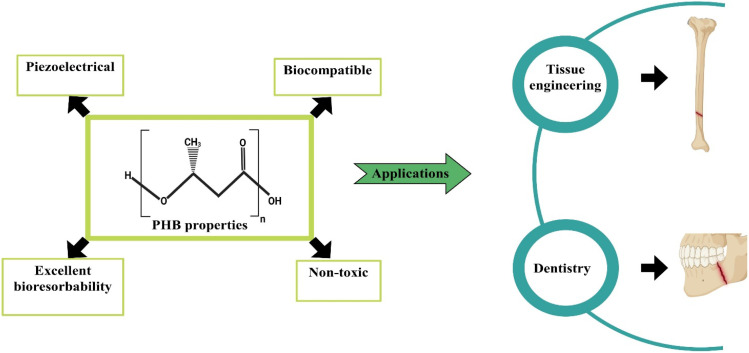
Structure, properties and applications of polyhydroxybutyrate (PHB).

Researchers are employing various strategies to efficiently produce Polyhydroxybutyrate (PHB) from different microbial strains.^72,73^ Poly(3-hydroxybutyrate) (PHB) stands out as a prominent member of the polyhydroxyalkonate (PHA) family, recognized for its molecular structure comprising homopolymerized (*R*)-3-hydroxybutyrate units. Exhibiting a molecular mass ranging from 200 to 20 000, PHB manifests as a microbial polyester that accumulates in the form of lipid inclusions. Within the microbial context, PHB's dynamic nature arises from the ester bonds formed between carboxyl groups of one monomer and adjacent hydroxy groups. This polyester displays isotactic characteristics attributed to the (R) stereochemical arrangement.^74^ The consideration of poly(3-hydroxybutyrate) (PHB) molecular mass holds primary importance in the realm of PHB applications, as it helps to maintain the mechanical characteristics of the polymer. The mechanical and thermal attributes of PHB are distinguished by notable features including elevated crystallinity within the range of 60% to 70%, a significant melting temperature of 175 °C, a commendable tensile strength falling between 30 and 35 MPa and an appropriate elasticity modulus of 3 GPa. It represents significance potential for biomedical applications, as shown in Fig. 4.^75^ PHB undergoes decomposition into 3-hydroxybutyric acid, a compound naturally occurring in human blood. In particular, 3-hydroxybutyric acid has been observed to enhance the calcium influx in cultured cells while concurrently suppressing cell death.^76^ In the natural environment, the degradation of PHB is facilitated by non-specific lipases and esterases. Therefore, it is reasonable to infer that those lipases and esterases play a substantial role in the *in vivo* degradation of PHB implants and associated medical devices. Additionally, it is remarkable that the mechanical and chemical properties of PHB-based materials remain unaffected by sterilization procedures.^77^

#### 2.3.2 Application of polyhydroxybutyrate in odontology

The prevalent approach to addressing dental issues involves the removal of damaged tissues, followed by their replacement with synthetic materials. The incorporation of bio glass into the solution resulted in increased electrical conductivity owing to its structure containing ionic groups. This, in turn, lead to a reduction in fibre diameters. The outcomes revealed that the addition of 15 wt% chitosan and 10 wt% bio-glass nanoparticles led to a fourfold increase in tensile strength compared to the control sample (pure PHB). Furthermore, an augmentation in the absorption of phosphorous and calcium ions was observed, attributed to the existence of nanoparticle bio-glass. Microscopic analysis depicted robust attachment of cells and increased proliferation of cells on the surface of scaffolds, underscoring the scaffold efficacy. These findings signify the potential of the PHB/chitosan/bio glass nanofiber scaffold in influencing stem cell behaviour for the development of odontoblast-like cells. The study provides valuable insights into advancing materials for dental applications that improved mechanical properties and enhanced ion absorption capabilities, ultimately promoting cellular attachment and proliferation on the surface of the scaffold.^78^

In a separate investigation, Khoroushi *et al.* conducted an assessment on the impact of an electro spun nanofiber scaffold composed of chitosan (CS), Polyhydroxybutyrate (PHB) and nanosized bioactive glass (nBG) concerning the proliferation and differentiation of stem cells sourced from shed teeth of human, aiming to induce the formation of odontoblast-like cells. Scanning electron microscopic images revealed robust cellular adhesion and pronounced proliferation of cells on the scaffold's surface, affirming the biocompatibility of the scaffolds and their non-toxic nature towards cells, and the results of real-time polymerase chain reaction analysis demonstrated a remarkable six-fold elevation in the level of expression of collagen type-1 genes and dentin sialophosphoprotein (DSPP), in contrast to the control group. These findings suggested that the engineered scaffold exhibited suitability for direct pulp capping applications.^79^

Zarei *et al.* examined the influence of electro spun scaffolds composed of Polyhydroxybutyrate and 1% carbon nanotubes (CNTs) on periodontal regeneration. The incorporation of 1% w/v CNTs into the polymer solutions facilitated the electrospinning process. The physical attributes of the resulting scaffolds were meticulously characterized using a universal testing machine, a scanning electron microscope, and a Fourier transform infrared spectrometer. *In vitro* assessments involved the cultivation of periodontal ligament stem cells (PDLSCs) on the scaffold over a period of 10 days, while *in vivo* investigations entailed the implementation of the scaffolds in a rat model for a duration of 5 weeks. These outcomes indicated the successful fabrication of polyhydroxybutyrate/carbon nanotube 1% scaffolds *via* electrospinning, exhibiting a fibrous conductive tissue structure reminiscent of the periodontal ligament (PDL). The tensile strength of the polyhydroxybutyrate/carbon nanotube 1% composite approached that of the PDL and displayed a significant enhancement in comparison with a pure polyhydroxybutyrate scaffold. The introduction of functionalized carbon nanotubes demonstrated improvements in scaffold bioactivity, *in vivo* tissue compatibility, wettability and *in vitro* cell viability. These findings suggested that the polyhydroxybutyrate/carbon nanotube 1% scaffold holds potential applications in periodontal tissue regeneration (PTR), offering a multifaceted approach that encompasses structural, mechanical and biological considerations.^80^

### 2.4 Silk fibroin (SF)

Silk fibroin is a fibrous protein extracted from the spider's silk and the silkworms. It possesses numerous favourable characteristics including outstanding compatibility with biological systems, degradability, minimal potential for eliciting an immune response, adjustable mechanical attributes and versatile applicability.^81^ The collective arrangement of silk fibroin can be broadly categorized into crystalline and non-crystalline regions. The structured and oriented β-sheets, cross β-sheets and α-helix configurations within the crystalline regions contribute significantly to the elevated strength observed in silk fibroin.^82^ Moreover, the exceptional functional versatility of silk fibroin allows for the creation of sponges, thin films, nanoparticles, hydrogels and an array of other biomaterials customized to meet diverse application requirements. Silk fibroin has been employed in numerous *in vivo* investigations involving cartilage, bone, skin tissue and other similar studies.^83^

Hydrogels derived from silk fibroin as the primary constituent, demonstrate exemplary tissue repair capabilities in accordance with diverse clinical application prerequisites. These hydrogels are characterized by diverse designs, manufacturing techniques and amalgamations with other materials.^84^ Extensively employed hydrogels derived from silk fibroin offer not only the benefits of a dependable and cost-effective source but also cover notable advantages, including variable mechanical properties, biocompatibility and regulated degradation and absorption.^85^

#### 2.4.1 Structure and properties of silk fibroin

**Fig. 5:**
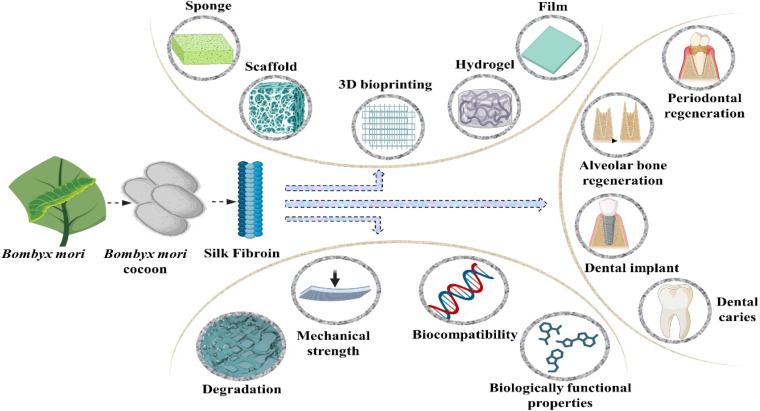
Different types of silk fibroin biomaterials, and their properties and applications.

Silk fibroin emerges as a promising biological polymer with distinctive characteristics for applications in bone tissue engineering. Combining silk fibroin with other materials to fabricate composite scaffolds promotes cellular activities such as cell differentiation, proliferation and adhesion. Furthermore, biomaterials based on silk fibroin can be transformed into diverse formats such as sponges, films, 3D structures, nanoparticles and hydrogels as depicted in Fig. 5. Silk fibroin, a natural protein, is primarily composed of amino acids such as serine, alanine, and glycine.^86^ In the case of spider silk, the process of spinning and exposure to air leads to its solidification, posing challenges in achieving substantial production of silk fibres. In contrast, the silk yield from a single silkworm cocoon is approximately ten times greater than that of spider's silk.^87^ It stands as the most extensively applicable silkworm, generating substantial quantities of silk characterized by enhanced quality. Within the *Bombyx mori* cocoons, impurities in the form of sericin constitute approximately 16.7–25%, while the protein silk fibroin comprises 5–3.3%. Silk fibroin, characterized by its partially-crystalline structure, is primarily used for its capacity to bear weight. In contrast, sericin a protein polymer lacking a defined structure serves as a material for gumming.^88^ Fibroin devoid of sericin has exhibited enhanced mechanical properties compared to fibroin enveloped in sericin, manifesting a 50% increase in tensile strength, a young modulus of 15–17 GPa and a breaking stress extending up to 19 percent. The enhancement of the silk degumming technique typically involves organic solvents and reagents, and scientists and researchers are actively engaged in refining procedures. The conventional soap degumming by the Marseilles technique has been superseded by the sodium carbonate (Na_2_CO_3_) degumming method, which is, currently, the frequently adopted approach due to its rapidity and cost effectiveness.^15^

The silk fibroin obtained from *Bombyx mori* cocoons is composed of two major chains, namely the heavy (H) chain with a molecular mass of 390 kDa and the light (L) chain with a molecular mass of 26 kDa. These chains are interconnected by a disulphide bond, forming the H–L complex. The cocoon's structural components consist of three polypeptides, namely the light chain, heavy chain and glycoprotein P25, present in a molar ratio of 6/6/1. The amino acid composition of the heavy chain includes serine (5.3%), valine (1.8%), alanine (30.3%), glycine (45.9%) and various other amino acid types (4.5%). This molecular arrangement and amino acid distribution characterize the intricate silk fibroin structure in *Bombyx mori*.^89^ The heavy (H) chain is primarily constituted by 60 to 75% repetitions of the glycine-x dipeptide motifs. Hydrophobic residues within these dipeptide repeats have the ability to form stable anti-parallel β-pleated sheets. Specifically, within the glycine-X dipeptide motif region, two hexapeptides with the sequences of peptide Gly-Ala-Gly-Ala-Gly-Tyr and Gly-Ala-Gly-Ala-Gly-Ser collectively contribute to 70 percent of this motif. The secondary structure arises from solutions of regenerated silk fibroin (RSF), exhibiting a combination of crystalline and amorphous structures.^90^ In its crystalline state, silk displays β-turns and forms a non-soluble structure characterized by folded β-sheets. Furthermore, in its amorphous state, silk exhibits helix-turns and comprises random coiling structures.^84^

#### 2.4.2 Application of silk fibroin in odontology

The pulp tissue, situated within the pulp cavity in teeth, is abundant in neural networks and blood vessels, playing a vital role in maintaining tooth health. Its heightened sensitivity to external stimuli is crucial. Regenerating damaged pulp, often stemming from infections and various factors, poses considerable challenges.^91^ Endodontic therapy is recommended for irreversible pulpal lesions, as it involves the extraction of the pulp it increases the risk of persistent dental infections.^92^ A preferable alternative to solid stents for intricate and irregular anatomy of the root canal system is an injectable system, it offers high sterility, low invasiveness and the capability to accommodate irregular voids.^93^ Promising alternative therapies involve the use of injectable hydrogels that replicate the viscoelastic properties of the native extracellular matrix in soft tissues. These hydrogels serve as carriers for delivering stem cells, contributing to the potential regeneration of dental pulp.^94^

Chen and colleagues innovatively created an injectable printing hydrogel derived from solidified regenerated silk fibroin, using carbamide and high-molecular-weight regenerated silk fibroin as raw materials. They successfully showcased the hydrogel commendable cytocompatibility with dental pulp mesenchymal stem cells, highlighting its capacity to facilitate their proliferation and growth.^95^ Similarly, hydrogels composed of fibroin and incorporating gelatinized cells have demonstrated favourable compatibility with human dental pulp stem cells. This was achieved through visible-light-induced cross-linking facilitated by vitamin B_2_ acting as a photo initiator.^96^ The proliferation of stem cells derived from human exfoliated deciduous teeth on silk sponges has been observed. However, the suitability of these scaffolds for repair of root canal necessitates additional *in vivo* investigation to validate their efficacy.^97^

Nie *et al.* developed an innovative scaffold using nano-hydroxyapatite mineralized silk fibroin (MSF) with the aim of reducing alveolar ridge resorption and promoting the rapid formation of new bones within tooth sockets. Their study included an investigation into the *in vitro* biocompatibility and osteogenic potential of MSF. Additionally, the research assessed the impact of preserving site using the mineralized silk fibroin graft in post-extractive sockets through *in vivo* experiments. The examination of the fabricated scaffold through EDX, SEM, XRD and FTIR spectroscopy analysis revealed a uniform distribution of granulated nanohydroxyapatite (nHA) crystals on the silk fibroin (SF) scaffold. Moreover, the hydrophilicity of the mineralized silk fibroin (MSF) scaffolds, as assessed by swelling and water contact angle ratio, exhibited higher-ranking characteristics compared to the plain silk fibroin scaffold. The presence of nanohydroxyapatite inorganic crystals demonstrated a positive influence on the MC3T3-E1 cells subjected to osteogenic differentiation, suggesting an enhancement in osteogenesis facilitated by the MSF scaffolds. Additionally, the application of MSF grafts resulted in increased bone formation and a reduction in alveolar bone resorption height following tooth extraction.^98^

### 2.5 Collagen

Collagen, an exceedingly plentiful fibrous protein, constitutes a predominant element within the extracellular matrix (ECM) across diverse animal species. Various forms of collagen can be isolated from fish, alligator and mammalian tissues. However, these native collagen variants exhibit insolubility in organic solvents, attributed to the inherent hydrophilic nature.^99^ This biological polymer serves as the principal constituent of connective tissues, comprising over 30% of the total protein mass within the organism. Functioning as an intracellular adhesive, it plays a crucial role in restraining tissues from undergoing excessive stretching or sustaining damage.^100^ Collagen is derived from both natural and synthetic reservoirs. Marine collagen emerges as a prospective natural source with the advantage of seemingly avoiding disease transmission.^101^ Harnessing the benefits of recombinant technology has led to the establishment of an alternative synthetic reservoir for collagen, ensuring high quality and freedom from animal-derived contaminants. This synthetic collagen is manufactured in diverse platforms including mammalian cells, insect cell culture, yeast and predominantly in plant cell cultures. However, its drawbacks of recombinant technology involve increased costs, reduced yields and the absence of essential cofactors or enzymes within these systems. For these regions, animal collagen remains the benchmark for utilization in both research and clinical domains.

#### 2.5.1 Structure and properties of collagen

**Fig. 6:**
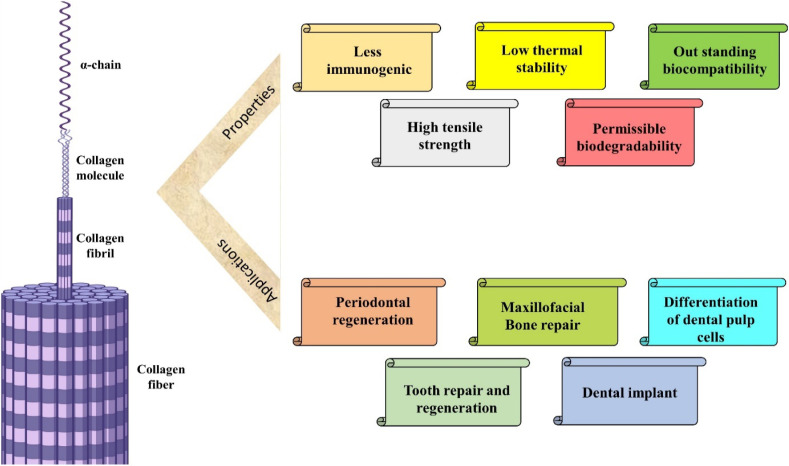
Structure of collagen with its properties and applications.

Collagen has gained attraction as the pivotal key component for biomaterials, owing to its outstanding biocompatibility, minimal immunogenicity and permissible biodegradability.^102^ Collagen plays a central role in upholding the mechanical resilience and biofunctionalities of connective tissues within the extracellular matrix (ECM). In pursuit of a biomimetic ECM substitute, a comparable collagen-like structure has been artificially constructed using supramolecular peptides.^103^ Collagen participates in cellular processes such as growth, migration and proliferation, intricately linked to physiological mechanisms of adaptation and tissue regeneration. However, natural collagen exhibits limited thermal stability and susceptibility to proteolytic degradation.^104^ Presently, a total of 29 unique collagen types have been recognized exhibiting different amino acid residue sequences, morphological structures, distribution patterns and biophysiological properties.^105^ The collagen superfamily serve various functions in distinct body tissues, imparting them with the capacity for a spectrum of biological activities. As an illustration, fibrillar collagen can furnish twisted three-dimensional frameworks for tissues or organs.^106^ Each collagen type features a triple helical strand, comprising three α-chains, which can be identical or distinct.^107^ The fundamental configuration of collagen comprises tripeptide sequences characterized by glycine–*X*–*Y*, wherein every triplet residue encompasses glycine. In this context, the *X* and *Y* positions are filled by proline and 4-hydroxyproline, constituting approximately 20% of the total composition. The secondary configuration is in the form of an α-helix, influenced by the steric hindrance phenomenon between proline and hydroxyproline residues located at the *X* and *Y* positions, respectively.^108^ The quaternary structure of collagen with its properties and applications in dentistry is depicted in Fig. 6.

#### 2.5.2 Applications of collagen in odontology

Collagen-based scaffolds have been employed in various hard tissues, specifically in applications related to maxillofacial bone, teeth and spinal cord repair.^109^ Teeth consist of three extensively mineralized tissues, namely dentin, cementum and enamel. It is well established that these mineralized dental tissues are marked by a limited capacity of self-regeneration.^110^ The regeneration of entire tooth is a very difficult and challenging task, that is why it requires the integration of biological, genetic and bioengineering methods.^111^

Lobat Tayebi and colleagues blended collagen and β-tricalcium phosphate (β-TCP) as the mineral phase to create a biomechanically stable scaffold, which is employed in the regeneration of teeth and craniomaxillofacial bones. This incorporation of collagen in the scaffolds resulted in enhanced cell proliferation and increased alkaline phosphatase (ALP) activity when co-cultured with dental pulp cells (DPCs) for a duration of 3 weeks. This scaffold manifests an interconnected pore, favourable mechanical properties and anisotropic microstructures attributed to the presence of collagen, highlighting its potential as a promising platform for bone tissue engineering.^135^

Jiang and collaborators created scaffolds using collagen/silk fibroin (CSF) through the application of Low-Temperature Deposition (LTD) by 3D printing technology. The impact of these scaffolds on the proliferation and differentiation of Dental Pulp Cells (DPCs) was subsequently assessed, considering their potential as substitutes for tooth repair in tissue engineering. The scaffold demonstrated a notable enhancement in cell adhesion and alkaline phosphatase (ALP) activity of DPCs, fostering the multi-layered growth of cells within the scaffold. These outcomes suggested the scaffold's potential application in generating the dental pulp complex.^130^

The recent findings on biologically active dental scaffolds have contributed to advancing research studies for the fabrication processes involved in dental tissue engineering. Additionally, these results have generated enthusiasm for the potential application of stem cell therapy in the regeneration of tooth tissue.^136^ Currently, dental pulp cells are the predominant cell type utilized in dental tissue engineering. However, the clinical implementation of dental pulp cells remains a formidable challenge.^137^ In addition to establishing standardized procedures for the isolation, validation, expansion, healing, storage and transportation of dental pulp cells, there are inherent responsibilities and considerations associated with the implantation of these cells.^138^ The list of some natural polymers with their properties and applications in dentistry is presented in Table 1.

**Table 1:** List of natural polymers with properties and applications in dentistry

S. no.	Natural polymers	Properties	Application in odontology	References
1	Collagen	It is the major component of extracellular matrix that interacts with cell, tissues and help in signal transduction	Dental caries, dentin remineralization, regenerative dentistry, root caries, dentin caries	112–116
2	Cellulose	One of the most utilized form of carbohydrate, useful for biomedical and medicinal purpose. It is hydrogen bonded and mostly cohesive in structure which make them insoluble in water	Oral health regeneration, dental caries, periodontitis, alveolar bone regeneration	117–120
3	Gelatine	It is a commercially significant polypeptide sourced from collagen and exhibit broad range application within the realm of both food and pharmaceutical industry	Alveolar socket defects, alveolar bone regeneration, orofacial bone regeneration, healing in the alveolar ridge, dental implants	121–125
4	Alginate	It is a natural polysaccharide obtained from seaweeds and has structural similarity to the ECM in tissues	Dental implants, periodontitis, enamel tissue engineering, dental enamel regeneration	126–129
5	Silk fibroin	It is a beta sheet structure made up of amino acid sequences mainly glycine, alanine and serine. It has good mechanical strength	Human dentine pulp regeneration, root canal fillers, dental implants, alveolar bone defects, periodontal tissue engineering	130–134

## 3 Modifications of natural polymers for biomaterial preparation

Natural polymers, with their inherent biocompatibility and biodegradability, are increasingly being explored for various biomedical applications. However, to enhance their properties and expand their potential uses, these polymers often require specific modifications. By altering their chemical structure, physical characteristics, and functional properties, natural polymers can be tailored to meet the stringent demands of modern biomaterials.^139^

In dentistry, the incorporation of calcium phosphate, hydroxyapatite, aluminium, bioactive glass, and nanoclays into biomaterials significantly enhances their properties. Calcium phosphate and hydroxyapatite are renowned for their excellent biocompatibility and osteoconductivity, promoting bone regeneration and integration with natural tissues. Aluminium, despite being a controversial choice due to potential toxicity, is utilized in specific controlled forms to enhance the mechanical strength and durability of dental materials. Bioactive glass stands out for its ability to bond with bone and stimulate cellular responses, thereby accelerating tissue repair. Nanoclays, including kaolin, bentonite, clay, and vermiculite, enhance the mechanical stability and thermal resistance of dental composites due to their high surface area, fine particle size, excellent absorption, plasticity, viscosity, and adsorption properties.^140^

### 3.1 LAPONITE® structure and composition

**Fig. 7:**
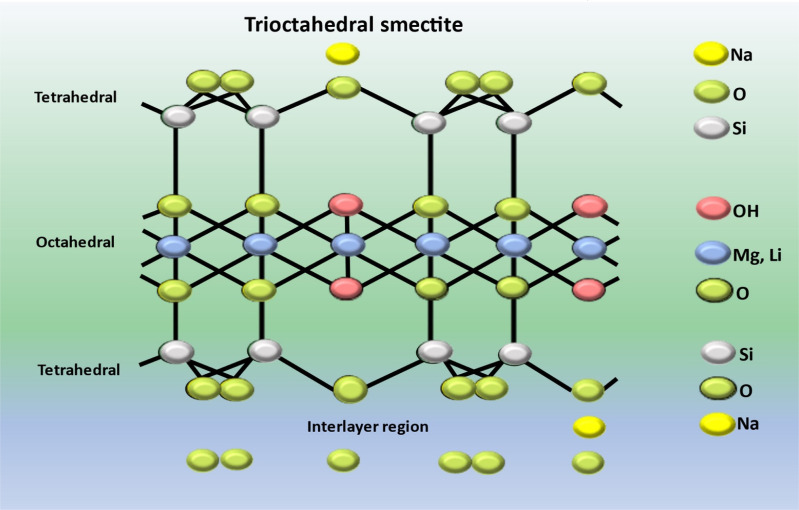
Structure of LAPONITE® (LAP).

LAPONITE RD® represents a gel forming variant of a synthetic sheet silicate possessing a crystalline configuration and chemical makeup related to the naturally occurring clay mineral known as hectorite.^141^ Circular, almost identical LAPONITE® crystals (with a chemical composition of Si_8_Mg_5.45_Li_0.4_O_24_Na_0.7_) consist of a single layer of octahedrally coordinated magnesium or aluminium oxide situated between two layers of tetrahedrally coordinated silica, as illustrated in Fig. 7. The unit cell carries an overall negative charge of approximately 700 electron charges, which is balanced out by the absorption of Na^+^ ions onto the crystal's surface within the interlayer space.^142,143^ Its disc-shaped structure possesses a negatively charged face and positively charged edges, enabling the formation of a “card castle” structure at approximately 2% mass concentration in an aqueous medium. When suspended in water, LAPONITE® undergoes gelation, forming a gel.^144^ LAPONITE® has the capacity to swell, absorbing significant amounts of water. Furthermore, it demonstrates high biocompatibility, anisotropic morphology and a substantial contact surface area. These distinctive characteristics render LAPONITE® suitable for the development of nanocomposites and diverse applications across various knowledge domains.^145^

#### 3.1.1 Application of laponite in dentistry

A recent investigation has revealed a promising direction in developing an injectable hydrogel that incorporates LAPONITE® (LAP), with the potential for application within the realm of dentistry. Hydrogels composed of alginate and LAPONITE®, enclosing dental pulp stem cells (dpsc) and vascular endothelial growth factor (VEGF), have demonstrated the ability to facilitate a continuous release of VEGF. This sustained release facilitates vascularization, thereby fostering the regeneration of tissues resembling dental pulp *in vivo*. Injectable RGD(arginine–glycine–aspartate)-alginate/LAP hydrogel microsphere was created, which encapsulates both human dental pulp stem cells (hDPSCs) and VEGF. These microspheres were fabricated by the electrostatic microdroplet method, yielding an average size ranging from 350 to 450 μm. By manipulating the LAPONITE® content, it was possible to adjust the rheological properties and degradation rate of the microsphere *in vitro*. The VEGF release from the RGD(arginine–glycine–aspartate)-Alg/0.5%LAP microspheres occurred steadily over a period of 28 days, maintaining the bioactivity of VEGF. Furthermore, the hDPSCs encapsulated within the microsphere were uniformly distributed, with a cell viability exceeding 85%. After 7 days, microspheres exhibited substantial deposition of extracellular matrix components such as fibronectin (FN) and collagen type I (Col-I). The presence of LAPONITE® in the system notably increased the expression of odontogenic-related genes in hDPSCs by day. Moreover, following subcutaneous implantation of tooth slices in a nude mouse model for one month, the RGD(arginine–glycine–aspartate)-Alg/0.5%LAP + VEGF microspheres laden with hDPSCs facilitated the regeneration of pulp such as tissues and the development of new micro vessels. These findings underscore the considerable promise of laponite-enhanced hydrogel microsphere in the context of vascularized dental pulp regeneration.^146^

A rat tooth extraction model was employed to evaluate the bone stimulating capability of the scaffold *in vivo*. Minimal instances of new bone formation were marked in both the control and silk fibroin/glycine groups. Conversely, the bio-oss and silk fibroin/glycine/LAPONITE® groups demonstrated differing levels of new bone formation. Encouraged by this finding, they conducted a quantitative analysis of new bone formation at that site of defects. The trabecular bone volume measurements for the bio-oss and silk fibroin/glycine/LAPONITE® (SF/Gly/LAP) groups surpassed those of the blank and silk fibroin/glycine groups by a considerable margin, suggesting enhanced osteogenic properties. Upon comparing bone mineral density (BMD) values among the various groups, it was evident that both the bio-oss and SF/Gly/LAP groups displayed more elevated BMD values than those of the remaining groups, with the SF/Gly/LAP group exhibiting the highest BMD value. In comparison to the bio-oss group, the SF/Gly/LAP group showcased enhanced new bone formation and mineralization. Conversely, the SF/Gly and blank groups exhibited more conspicuous bone defects at identical sites. Taken together, these results underscore the efficacious role of SF/Gly/LAP in stimulating osteogenesis, thus positioning it as a promising contender for alveolar bone regeneration at tooth extraction sites. Henceforth, this research suggested a hopeful method for regenerative repair of alveolar bone post tooth extraction. The amalgamated scaffold material, with its homeostatic and osteogenic properties, along with shape memory characteristics, shows potential to improve the success of dental implants and contribute positively to overall oral health.^147^

Xu and colleagues conducted a study examining the impacts of polycaprolactone (PCL) and LAPONITE® (LAP)-incorporated nano silicate both on periodontal ligament cells (PDLCs) *in vitro* and on periodontal regeneration *in vivo*. A nanofiber-based membrane composed of PCL/LAP was synthesized *via* the electrospinning technique. To characterize the nanofiber-based membrane PCL/LAP, methods such as energy dispersive X-ray Spectroscopy (EDS), Scanning Electron Microscopy, tensile testing and Inductively Coupled Plasma Mass Spectroscopy (ICP-MS) were used. Subsequently, the proliferation and osteogenic differentiation of periodontal ligament cells on the polycaprolactone/LAPONITE® nanofiber-based membrane were assessed. Furthermore, an *in vitro* coculture system comprising PDLCs and macrophages was utilized to investigate the immunomodulatory properties of the PCL/LAP nanofiber-based membrane. A nanofiber-based membrane comprising PCL/LAP was surgically implanted into a rat's calvarial bone and periodontal defects, followed by assessment of its regenerative efficacy using microcomputed tomography (micro-CT) and histological examination. The PCL/LAP nanofiber-based membrane exhibited favourable biocompatibility and bioactivity, promoting the proliferation and osteogenic differentiation of periodontal ligament cells. Additionally, it facilitated the generation of anti-inflammatory N2 neutrophiles and promoted tissue remodelling, while also modulating inflammatory responses and promoting M2 macrophage polarization through its immunomodulatory effects on PDLCs. *In vivo* experiments demonstrated that the PCL/LAP nanofiber-based membrane effectively expedited the repair of rat calvarial bone defects and promoted periodontal regeneration. Furthermore, *in vitro* studies revealed that the incorporation of LAP nano silicate into the PCL membrane facilitated osteogenesis and modulated the immune response of periodontal ligament stem cells. These findings suggest that the composite membrane holds great promise as a biomaterial for periodontal regeneration therapy.^148^

### 3.2 Structure and properties of montmorillonite (MMT)

Montmorillonite (MMT) represents an economic natural clay mineral, constituting a vital element in sedimentary rock and soil derived from layered silicates. Montmorillonite, characterized by the molecular formula (Na, Ca)_0.33_ (Al, Mg)_2_(Si_4_O_10_) (OH)_2_·*n*H_2_O^149^ represents a biodegradable monoclinic silicate mineral featuring a layered “sandwich” sheet structure comprising aluminium–oxygen octahedrons and silicon–oxygen tetrahedrons. Recognized as a conventional clay mineral, it finds extensive applications in reinforcing the mechanical properties of bone scaffolds.^150^

Montmorillonite clay is an indigenous hydrous aluminium silicate clay categorized as 2 : 1 phyllosilicate within the clay class. These clays, falling under the aforementioned classification, are typically recognized as stratified silicates. This clay exhibits a stratified structure comprising layers of silicate platelets with a thickness of approximately 1 nanometre and lateral dimensions ranging from 300 nanometres to a few microns. Each individual platelet is composed of a central octahedral alumina sheet sandwiched between two outer silica tetrahedral sheets. The tetrahedral sheet predominantly consists of silica, while the octahedral sheet encompasses various elements such as Al, Mg and Fe. The arrangement of these layers results in a systematic van der Waals gap, referred to as the interlayer or gallery.^151^ The structural stability of montmorillonite, characterized by intermolecular forces, involves van der Waals forces and electrostatic effects.^152^ Simultaneously, the intramolecular interaction force serves to maintain the stability of the intralayer domain in a constant state, creating a conductive environment for the incorporation of biomolecules such as anti-inflammatory drugs or the intercalation of modifiers such as polymer molecules.^153^ In recent research, multiple investments have demonstrated that polymer matrices incorporating nanofibers exhibit favourable biological properties alongside adequate mechanical characteristics.^154^

As an inherent clay nanomaterial, montmorillonite not only possesses a substantial specific surface area, significant aspect ratio, elevated rigidity and favourable processability but also exhibits effective compatibility with polymeric matrix materials, achieved through both physical and chemical interactions.^155^ Studies have indicated that a relatively low proportion of nano montmorillonite filler suffices to markedly enhance the mechanical characteristics of composite materials.^156^

#### 3.2.1 Applications of montmorillonite (MMT) in odontology

In a study, montmorillonite with a hydroxyapatite layer and calcium montmorillonite were utilized as fillers in dental composites, aiming to enhance the remineralization potential. Silane modification was employed to improve adhesion to methacrylic resin. The efficacy of filler preparation was verified by various techniques including SEM, TEM, X-ray, EDS, FTIR spectroscopy and nitrogen adsorption/desorption. The successful incorporation of calcium, hydroxyapatite mineralization on montmorillonite surfaces and particle silanization were achieved. The dental composite properties were assessed, encompassing the degree of conversion, the depth of cure, flexural and comprehensive strength, mass stability, and remineralizing potential, gauged by calcium ions released under simulated oral conditions. Both calcium and hydroxyapatite forms of montmorillonite demonstrated the ability to release Ca^2+^, albeit with a significantly higher remineralizing potential absorbed in the calcium form. Giving its proficiency in incorporating and releasing calcium ions, further investigation is warranted to explore their application of calcium-rich montmorillonite as an active filler in dental composites, showcasing promising properties for dental applications.^157^

Research aimed to develop a low-shrinkage dental composite incorporating nano clay fillers (montmorillonite Closite®-MMT), and assessed its cytotoxicity and physio-mechanical characteristics. Four dental composites were synthesized using a consistent organic matrix (Bis-GMA/TEGDMA, 30 wt%), with different concentrations of nano clay fillers (BaSi, SiO_2_ and MMT): 93.8/6.2/0, 89.1/5.9/5, 86.7/5.8/7.5 and 84.4/5.6/10 (E0, E5, E7.5 and E10: representing 0%, 5%, 7.5% and 10% of MMT nanoclays, respectively). Multiple properties were evaluated, including *in vitro* cytotoxicity, flexural strength, elastic modulus, volumetric shrinkage, water sorption, water solubility and hygroscopic expansion. SEM technique was implemented for topographical characterization. Statistical analysis was performed on the data. The results indicated that MMT nanoclays had no significant impact on cytotoxicity. The E5 and E7.5 groups exhibited a significant reduction in polymerization shrinkage while maintaining overall physio-mechanical properties. Incorporating 5% and 7.5% wt. of MMT nanoclays facilitates the creation of dental composites with low cytotoxicity and diminished polymerization shrinkage, without compromising other essential characteristics such as flexural strength, elastic modules, water sorption, water solubility and hygroscopic expansion. These findings suggest that the incorporation of MMT nanoclays presents a viable strategy for formulating new dental composites with potential clinical applications.^183^

Current clinical practices used sealants for filling, but these lack sustained fluoride release and recharge capability. Research introduces a novel fluoride-montmorillonite nanocomposite resin, aiming to provide prolonged fluoride release through the strong fluoride adsorption by montmorillonite. The successful integration of the polymer into the interlayer structure was confirmed by X-ray diffractometry, thermogravimetric analysis and FTIR spectroscopy. Mechanical properties such as hardening depth, viscosity, diametral tensile strength, effective fluoride ion release and recharge show potential for the prevention of dental caries. Additionally, the non-cytotoxic nature of the material was affirmed through the water-soluble tetrazolium salt (WST-I) test. The study anticipates that this fluoride containing composite resin could emerge as a promising clinical alternative in the near future, offering an enhanced approach to preventing dental caries.^182^ Some organic and inorganic fillers are listed in Table 2, along with their properties and applications in odontology. Hardness, wear resistance and mechanical strength were evaluated for the developed composite resin. Simulation of the oral environment is demonstrated.

**Table 2:** Organic and inorganic fillers with properties and applications

Sl no.	List of organic/inorganic fillers	Properties	Application in odontology	References
1	Calcium phosphate	Calcium phosphate materials exhibit compositional similarity to bone and share bioactivity and osteoconductive properties. Various formulations of calcium phosphate materials, including composites, coatings and cements, find widespread applications in diverse medical and dental contexts	Cancer bone treatment, dental implant, periodontal and alveolar bone regeneration, dentin regeneration, sinus lift, tooth replacement	158–163
2	Hydroxyapatite	Hydroxyapatite demonstrates advantageous bioactive and osteoconductive characteristics, facilitating prompt bone formation within a host organism and robust biological adherence to bony tissues. Despite these benefits, it exhibits diminishing mechanical strength and facture toughness	Endodontic treatment, tooth whitening, dental implants, orthodontics, dental composite, periodontal tissue regeneration	164–170
3	Aluminium	Alumina, alternatively known as aluminium oxide, represents the sole solid oxide manifestation of aluminium (Al_2_O_3_)	Maxillofacial implant, dental implants, dental pulp tissue, dental fillers	171–174
4	Bioactive glass	Within the spectrum of biomaterials, bio glasses stand out as particularly intriguing owing to their capacity to generate a highly responsive carbonate hydroxyapatite layer. Bio glass, contributing a family of bioactive glasses, is composed of silicon dioxide, calcium oxide, sodium oxide and phosphorous pentoxide	Periodontitis and dental implants, orthodontics, endodontics, oral and maxillofacial surgery, oral care products	175–179
5	Nanoclays	It is layered mineral silicate material processing biocompatibility, demonstrating enhanced physical and mechanical characteristics	Periodontitis, alveolar bone regeneration, periodontal regeneration, Craniofacial regenerative medicine, endodontics, dental caries	14, 147, 148, 180–182

## 4 Conclusion

This review paper primarily centres on providing foundational insights into natural polymer material-based bone regeneration, aiming to furnish researchers with a comprehensive understanding of the potential applications of these materials within the oral domain. Natural polymers such as chitosan, collagen and Polyhydroxybutyrate have demonstrated significant potential in enhancing the efficacy and biocompatibility of regenerative treatments and shown promising results in promoting cell adhesion, proliferation, and differentiation, essential for effective bone healing and regeneration. The integration of these biopolymers with bioactive molecules and their ability to form scaffolds mimicking the natural extracellular matrix further underscores their suitability for clinical applications. The biodegradable products of natural polymers are usually non-toxic and their degraded products can participate in various signaling pathways, similar to the roles of lithium and magnesium ions found in LAPONITE® nanoclays. Additionally, different types of montmorillonites, such as quaternary ammonium salts, possess broad-spectrum antimicrobial properties. These characteristics make them suitable for synthesizing nanocomposites that can address various health-related issues. However, despite these encouraging developments, challenges such as optimizing polymer properties, ensuring consistent quality, and overcoming regulatory hurdles remain. The inherent complexity associated with alveolar bone regeneration underscores the significance of advancing bioactive materials as a critical aspect in overcoming the inherent difficulties in this intricate regenerative procedure. The heightened interest in bio-nanocomposites stems from their improved material characteristics facilitated by the incorporation of nano-reinforcements. It is crucial to acknowledge the drawbacks associated with nanomaterials, such as their susceptibility to toughness issues. However, this limitation can be mitigated by adopting a hierarchical design approach that incorporates multiple length scales, thus yielding scaffold materials with enhanced strength and resilience. Additionally, nanomaterials possess the propensity to elicit adverse effects, including potential dispersion throughout the body or accumulation in organs due to their nanoscale dimensions, akin to biological molecules and viruses, and addressing these concerns necessitates further research efforts to develop strategies for potential future applications. Future research should focus on addressing these issues, as well as conducting long-term clinical studies to validate the safety and effectiveness of these natural polymer-based approaches in alveolar bone regeneration. Overall, the continued exploration and innovation in this field hold great promise for improving patient outcomes in dental and orthopaedic applications.

## Data availability

No primary research results, software or code have been included and no new data were generated or analysed as part of this review.

## Conflicts of interest

There are no conflicts to declare.
